# Alkylglycerol Monooxygenase

**DOI:** 10.1002/iub.1143

**Published:** 2013-02-25

**Authors:** Katrin Watschinger, Ernst R Werner

**Affiliations:** Division of Biological Chemistry, Biocenter, Innsbruck Medical UniversityInnsbruck, Austria

**Keywords:** tetrahydrobiopterin, ether lipid, alkylglycerol monooxygenase, E.C.1.14.16.5, glyceryl ether monooxygenase

## Abstract

Alkylglycerol monooxygenase (E.C. 1.14.16.5), also called glyceryl ether monooxygenase, is a tetrahydrobiopterin-dependent enzyme. It is the only enzyme known to cleave the ether bond of alkylglycerols and lyso-alkylglycerol phospholipids, including lyso-platelet activating factor. Although it has been first described already in 1964, it was not possible so far to purify the protein. It took until 2010 to assign a sequence to this labile integral membrane enzyme by bioinformatic selection of candidate genes, recombinant expression of these, and sensitive monitoring of the enzymatic activity by a fluorescence-based assay. The sequence shows no significant similarity with the other known tetrahydrobiopterin-dependent enzymes but contains the fatty acid hydroxylase protein motif signature. Proteins containing this signature are all labile and catalyze reactions similar to the alkylglycerol monooxygenase reaction. They are thought to use a di-iron centre for catalysis. Site directed mutagenesis of alkylglycerol monooxygenase defined a region of the active site and a conserved glutamate residue important for tetrahydrobiopterin interaction. Current research now focuses on defining a physiological role of this enzyme which occurs not only in mammals but also in commonly used model organisms such as zebrafish and the nematode *Caenorhabditis elegans*. © 2013 IUBMB Life 65(4):366–372, 2013.

## INTRODUCTION

In the 1960s, following the characterization of the structure of the tetrahydrobiopterin cofactor of phenylalanine hydroxylase (1), two further tetrahydrobiopterin-dependent aromatic amino acid hydroxylases were soon detected (2, 3). It then took until the 1980s to describe the three isoforms of the tetrahydrobiopterin-dependent nitric oxide synthases (4). Already in 1964, however, an enzyme system for the tetrahydropteridine-dependent cleavage of glyceryl ethers was first characterized (5).

Ether lipids (alkylglycerols, glyceryl ethers) form a diverse class of glycerol-based lipid and phospho-lipid compounds, which are less well studied than the corresponding ester lipids (acylglycerols). Ether lipids are essential to protect the eye from cataract, to allow the development of functional semen, and to enable the correct development of brain structures (6). Platelet activating factor (PAF), a well described potent inflammatory mediator (7) is an example of an alkylglycerol phospholipid signaling molecule. Ether lipids have been tested for their therapeutic potential in stimulating the immune system including activation of macrophages, stimulation of cytokine production, and interference with the PAF signaling pathway. They have anticancerogenic effects and have been shown to be able to transiently open the blood brain barrier and thereby give cytotoxic drugs access to the brain (8).

While the sequences of the other tetrahydrobiopterin-dependent enzymes which are all soluble proteins were then soon analyzed, the enzymes expressed in recombinant form and crystal structures solved (4), comparatively slow progress was made with the alkylglycerol monooxygenase, presumably because this labile membrane protein till today cannot be purified. Alkylglycerol monooxygenase is the only enzyme known to cleave the ether bond in alkylglycerol ether lipids. Since these are a class of biologically very important compounds, we are convinced that research on alkylglycerol monooxygenase function holds a great potential to unravel physiological mechanisms which might explain thus far poorly understood observations in tetrahydrobiopterin deficiency and tetrahydrobiopterin treatment. In this review, we summarize the current knowledge on the biochemistry of alkylglycerol monooxygenase, assays to measure its activity, its properties, and its occurrence.

## THE ALKYLGLYCEROL MONOXYGENASE REACTION

**FIG 1:**
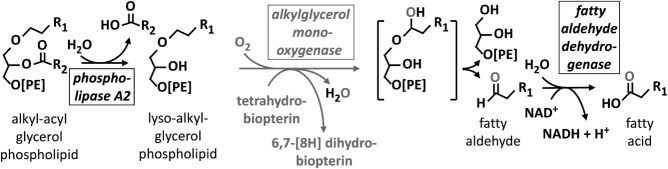
Role of alkylglycerol monooxygenase in the degradation of ether lipids. Alkylacylglycerol phospholipids resident in the membrane are cleaved by a PLA2 to become lyso-alkylglycerol phospholipids which are then substrates for alkylglycerol monooxygenase. Cleavage of the ether bond by molecular oxygen and the cofactor tetrahydrobiopterin (both marked in red online) is thought to occur via a hydroxylation and spontaneous or enzyme-assisted rearrangement of the resulting semiacetal into the free glycerol derivative and a fatty aldehyde which is then further oxidized to the corresponding acid by fatty aldehyde dehydrogenase, an NAD^+^ dependent enzyme. The cofactor tetrahydrobiopterin leaves the alkylglycerol monooxygenase reaction as 6,7-[8H]-dihydrobiopterin. PE stands for phosphoethanolamine, an example for the various phospholipids cleaved by the enzyme. R1 and R2 are representative lipid side chains which are saturated linear hydrocarbon chains, sometimes with one or more nonconjugated double bonds not adjacent to the ether linkage. Typical side chains have in total 16 or 18 carbon atoms. A range of 12–20 carbon atoms is accepted by alkylglycerol monooxygenase. [Color figure can be viewed in the online issue, which is available at wileyonlinelibrary.com.]

Figure 1 shows the alkylglycerol monooxygenase reaction in the context of the degradation of an example alkylglycerol phospholipid. First, the acyl group at *sn*2 of the glycerol backbone is cleaved off by a phospholipase A2 (PLA2). This step is reversible in that the free hydroxyl group at *sn*2 may be reacylated back to 1-*O*-alkyl-2-acyl glycerophospholipids by acyl transferases. The lyso-alkylglycerol phospholipid product of the PLA2 reaction is a substrate of alkylglycerol monooxygenase which hydroxylates the ether-bound fatty alcohol side chain at the carbon atom adjacent to the ether bond in a tetrahydrobiopterin-dependent mixed function oxygenase reaction. The resulting hemiacetal is chemically instable and rearranges to a fatty aldehyde and the corresponding glycerol derivative. It is not known whether this rearrangement occurs only spontaneously or whether it is facilitated by an additional, unidentified enzyme. Tetrahydrobiopterin leaves the reaction as the quinoid 6,7-[8H] dihydrobiopterin. This quinoid dihydrobiopterin is recycled back to tetrahydrobiopterin by dihydropteridine reductase (not shown in Fig. 1) (4, 5, 9). Although this has not been addressed experimentally so far it seems reasonable to assume that the spontaneous dehydratation of 4a-hydroxytetrahydrobiopterin, the initial product of the tetrahydrobiopterin-dependent hydroxylation, to the quinoid 6,7-[8H] dihydrobiopterin is assisted by carbinolamine dehydratase like in the phenylalanine hydroxylase reaction (10, 11). The toxic fatty aldehyde product of the alkylglycerol monooxygenase reaction is then further oxidized to the corresponding fatty acid by fatty aldehyde dehydrogenase (gene symbol ALDH3A2) (12). Both, the alkylglycerol monooxygenase and the fatty aldehyde dehydrogenase reaction are irreversible. Comparison of the activities of these two enzymes in mouse tissues showed that fatty aldehyde dehydrogenase activities (13) are always at least 10 times higher than alkylglycerol monooxygenase activities (14). This relationship ensures that the toxic fatty aldehyde products of the alkylglycerol monooxygenase reaction are effectively converted to the less toxic fatty acids in all tissues examined.

## SUBSTRATE SPECIFICITY AND POSITION IN ETHER LIPID METABOLISM

Substrate specificity of alkylglycerol monooxygenase has been studied in detail using rat liver microsomes, and these studies have been reviewed in 1998 (9). The substrate specificity is wide, but a few elements are essential. The ether-linked fatty alcohol residue at *sn*1 must not have a double bond adjacent to the ether linkage. Thus, vinyl ether lipids such as plasmalogens and lyso-plasmalogens are no substrates. The chain length of the fatty alcohol residue at *sn*1 must be between 12 and 20 carbon atoms to provide a good substrate. The hydroxyl group at *sn*2 must be free like in lyso-lipids, already a methoxy residue results in much lower activity. Compounds with acetyl or acyl groups at *sn*2 are not substrates. At *sn*3, a free hydroxyl group, or common phospholipid substituents such as phosphoethanolamine or phosphocholine are readily accepted. The carbon 3 may even be missing, that is, alkylglycols are good substrates (15). Likewise, glycerols with an ether-linked fatty alcohol only at *sn*2 and two free hydroxyl groups at *sn*1 and *sn*3 are also good substrates (9). Interestingly alkylglycerols with a phosphate group at *sn*3 such as in the ether lipid biosynthetic intermediate 1-*O*-alkyl-*sn* glycerol-3-phosphate are no substrates (16).

**FIG 2:**
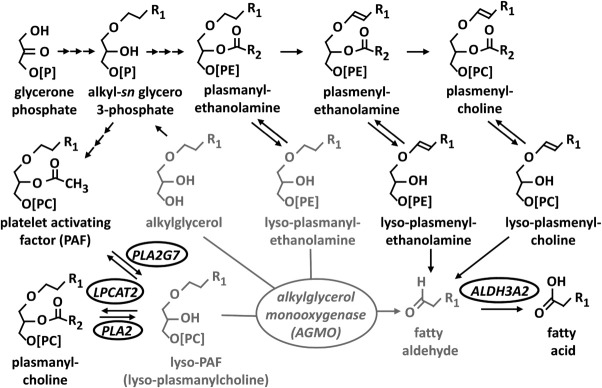
Simplified scheme of ether lipid metabolism and the central role of alkylglycerol monooxygenase in ether lipid degradation as suggested by its wide substrate specificity. Ether lipids are synthesized in the body in a complex set of reactions from glycerone phosphate (dihydroxyacetone phosphate). The first ether bond containing intermediate, alkyl-*sn*-glycero-3-phosphate, is an important branching point leading to all plasmanyl (harboring a saturated carbon side chain or one or more nonconjugated double bonds distal of the ether linkage which is connected to the *sn*1 position of glycerol via an ether bond) and plasmanyl (with a vinyl ether at the *sn*1 position of glycerol) species as well as to PAF. Lyso-PAF and lyso-plasmanyl lipids as well as alkylglycerols contained in nutrition (all drawn in red online) can then be degraded by alkylglycerol monooxygenase to a fatty aldehyde. In the so-called remodeling pathway, the PAF is synthesized via lyso PAF released from plasmanylcholine by action of a PLA2. Lyso PAF is in equilibrium with PAF by acetylation carried out by a specific acetylase (LPCAT2), and deacetylation by a specific PLA2 type enzyme (PLA2G7). By irreversible degradation of lyso-PAF, alkylglycerol monooxygenase might be capable of switching off the PAF signal (see text for details). PE stands for phosphoethanolamine, PC for phosphocholine, P for phosphate. R1 and R2 are representative lipid side chains which are saturated linear hydrocarbon chains, sometimes with one or more nonconjugated double bonds not adjacent to the ether linkage. Typical side chains have in total 16 or 18 carbon atoms. [Color figure can be viewed in the online issue, which is available at wileyonlinelibrary.com.]

From this substrate specificity a central role of alkylglycerol monooxygenase in the degradation of alkylglycerols can be deduced which is displayed in a simplified scheme in Fig. 2. For full details of the ether lipid metabolic reactions see ref.^17^. A variation of alkylglycerol monooxygenase activity might have important physiological consequences for example for the action of the PAF. The biological activity of this inflammatory mediator is known to be primarily controlled by the so-called remodeling pathway (7). The important, regulated trigger for PAF synthesis is the release of its lyso form from plasmanylcholine by PLA2. This is then converted to the active PAF by a specific acetylase, that is, alkylglycerophosphocholine *O*-acetyltransferase (E.C. 2.3.1.67, gene symbol LPCAT2, (18)). The same enzyme is also capable of acylating the lyso PAF to the plasmanylcholine membrane lipid (18). Cleavage of PAF to its lyso-form is carried out by 1-*O*-alkyl-2-acetylglycerophosphocholine esterase, E.C. 3.1.1.47, gene symbol PLA2G7, (19)). As lyso-PAF is a good substrate of alkylglycerol monooxygenase (20), it can be assumed that this enzyme has an anti-inflammatory action by switching off the PAF signal by irreversible degradation of its lyso form (Fig. 2).

## PURIFICATION ATTEMPTS AND SEQUENCE ASSIGNMENT

Several laboratories have described properties of alkylglycerol monooxygenase in rat liver microsomes, but no purification followed by sequencing of the protein was ever described. Ishibashi and Imai (21) developed protocols for the solubilization of the enzyme and prepared a resin for affinity chromatography by coupling 1-*O*-hexadecyl-*sn* glycerol to sepharose 4B. In a four step purification protocol, using 6-aminohexyl sepharose, diethylaminoethyl cellulose, sucrose density centrifugation, and hexadecylglycerol affinity chromatography, a single 45.0 kDa band in sodium dodecyl sulphate polyacrylamide disc gel electrophoresis was obtained (22). The authors did, however, not report on results of sequencing of this protein which is somewhat smaller than the 51.7 kDa calculated molecular weight deduced from the rat alkylglycerol monooxygenase sequence.

We failed in our attempts to purify the protein from rat liver microsomes (14). To assign the sequence, we selected 10 candidate genes and expressed them recombinantly in Chinese hamster ovary cells. One of the selected candidate genes, TMEM195, resulted in a pronounced, tetrahydrobiopterin-dependent alkylglycerol monooxygenase activity (14). TMEM195 is a predicted membrane protein with previously unknown function derived from the mammalian genome characterization efforts. The sequence has no homology to the other known tetrahydrobiopterin-dependent enzymes and contains the fatty acid hydroxylase motif. This motif occurs in integral membrane proteins which carry out hydroxylation reactions of lipid substrates like alkylglycerol monooxygenase does. All fatty acid hydroxylase motif containing enzymes are labile and cannot be purified (23). The fatty acid hydroxylase motif is characterized by the occurrence of eight conserved histidines (24), all of which are essential for alkylglycerol monooxygenase activity (14). Fatty acid hydroxylase motif containing proteins are thought to catalyze their hydroxylation reactions using a di-iron centre (23). In addition to the lacking sequence homology, the nature of the iron used distinguishes alkylglycerol monooxygenase from the other two classes of tetrahydrobiopterin-dependent enzymes, the aromatic amino acid hydroxylases which use a single non-hem iron, and the nitric oxides synthases which use a heme iron centre for oxygen activation (reviewed in ref.^4^). None of the other fatty acid hydroxylase motif containing proteins is known to require the tetrahydrobiopterin cofactor, and none of these proteins contain a glutamate in the position of the sequence where the conserved glutamate important for tetrahydrobiopterin interaction is located in alkylglycerol monooxygenase sequences (25).

## OCCURENCE

The alkylglycerol monooxygenase gene occurs in animals ranging from *Caenorhabtidis elegans* and various fish to mammals including rodents, dogs, chicken, chimpanzees, and humans. Early experimental data confirm the presence of alkylglycerol monooxygenase in the liver of several mammalian species (26). Interestingly, an alkylglycerol monooxygenase sequence is lacking in the *Drosophila melanogaster* genome. In *Leishmania donovani*, an alkylglycerol cleaving activity was detected which however proved not to be dependent on tetrahydrobiopterin but uses NADPH as cofactor (27). In line with these observations, no sequence with high similarity to alkylglycerol monooxygenase is found in the *Leishmania* proteome databases.

In mice and rats, tissue distribution of the enzymatic activity was investigated showing highest activities in the liver, followed by intestine and stomach, testis, fat tissues, bladder, and brain. No activity could be found in heart and skeletal muscle (14, 28). These data are in good agreement with gene expression data (14). Within the cell, alkylglycerol monooxygenase was located in the endoplasmic reticulum when expressed in fusion with green fluorescent protein (14).

## ACTIVITY ASSAYS FOR ALKYLGLYCEROL MONOOXYGENASE

Over the decades different assays to quantify alkylglycerol monooxygenase activity have been established by different groups. Early assays used tritiated or ^14^C labeled substrates followed by lipid extraction, thin-layer chromatography, and counting of the radioactive signal (26) or spectrophotometrically quantified the resulting aldehyde after derivatization with *p*-nitrophenylhydrazone (5). Another assay is based on NADH consumption by dihydropteridine reductase, which reduces the quinoid dihydro form of the cofactor back to its tetrahydro form and monitored this decrease spectrophotometrically at 340 nm (29). A drawback of this strategy is the interference by other NADH consuming reactions in crude extracts. Armarego and Kosar-Hashemi adapted an assay previously used for phenylalanine hydroxylases, which spectrophotometrically measures the conversion of tetrahydrobiopterin to quinoid dihydrobiopterin (29, 30). To obtain a more sensitive, nonradioactive assay, a fluorescent, pyrene-labeled alkylglycerol was synthesized and used as substrate. This 1-*O*-pyrenedecyl-*sn* glycerol is readily accepted by the enzyme (28). The pyrenedecanal product of the alkylglycerol monooxygenase reaction is converted to pyrenedecanoic acid by fatty aldehyde dehydrogenase present in excess in all tissues examined so far. In case of recombinant expression of alkylglycerol monooxygenase, or in case of attempts to purify the enzyme, however, fatty aldehyde dehydrogenase has to be added to the reaction mixture to ensure efficient formation of the stable product pyrenedecanoic acid from the initial product pyrenedecanal (14). Pyrenedecanoic acid is readily separated by high performance liquid chromatography (HPLC) from the substrate and sensitively quantified by fluorescence detection. (28).

## MECHANISTIC PROPERTIES OF ALKYLGLYCEROL MONOOXYGENASE

Due to the difficulties in purifying alkylglycerol monooxygenase most studies investigating properties of alkylglycerol monooxygenase which are available to date have been performed with tissue homogenates of rat liver. As alkylglycerol monooxygenase is highly expressed in this organ and resides in the membrane fraction, rat liver microsomes were prepared and used in various assays. Using rat liver microsomal preparations, Kaufman et al. (15) could show that the enzyme is not inhibited by carbon monoxide. This allowed the conclusion that alkylglycerol monooxygenase is not a cytochrome P450-dependent hydroxylase. Phenanthroline, a strong iron chelator, but not ethylene diamine tetraacetic acid was able to abolish alkylglycerol monooxygenase activity in a similar concentration range as for phenylalanine hydroxylase whilst leaving nitric oxide synthase totally unaffected. This indicated that a non-heme iron might be required for the catalysis in alkylglycerol monooxygenase (31).

Dependency on cofactors was also thoroughly investigated by different groups. After its first description in 1964 as a tetrahydropteridine-dependent enzyme (5), the absolute dependence on the tetrahydropterin cofactor was confirmed by Soodsma et al. in 1972 (32). The authors were also able to show that ammonium ions and glutathione were able to stimulate the reaction and that the pH optimum for the enzyme is approximately 9. Rock et al. (33) found that catalase had a stimulatory effect on alkylglycerol monooxygenase by protecting on the one hand the pterin cofactor from H_2_O_2_ but on the other hand also the enzyme itself from being attacked by this oxidizing agent. Kaufman and coworkers confirmed in 1990 that the activity of the enzyme is absolutely specific to tetrahydropterins while NADPH, NADH, glutathione, and reduced dichlorophenolindophenol were absolutely inefficient to promote ether lipid cleavage. Among the pterins, tetrahydrobiopterin and 6-methyl tetrahydropterin were the best cofactors, followed by 6,7-dimethyl tetrahydropterin and tetrahydrofolate. Kaufman and colleagues also found that catalase and ammonium were able to further stimulate the reaction (15). Influence of the nature and stereochemistry of the tetrahydropterin side chain on catalysis was analyzed in some more detail by a fluorescence HPLC-based assay. 6*R*-Tetrahydrobiopterin rather than 6*S*-tetrahydrobiopterin was found to be the active form of the cofactor. 6*RS*-6,7-Dimethyltetrahydropterin and 6*RS*-tetrahydroneopterin were equally able to elicit catalysis while 6*RS*-tetrahydrodictyopterin and 6*RS*-6-methyltetrahydropterin displayed only about half the efficiency (28). It was also confirmed that other reductants like glutathione, FADH_2_, or l-ascorbic acid were not able to take over the function of the tetrahydropterin cofactor.

The stoichiometry of tetrahydropterin oxidized to ether lipid cleaved was found to be 1:1 (34) or close to 1:1 (15), or 1:1 with the assumption that only half of the added *R*,*S* mixture of the 6-methyltetrahydropterin added was enzymatically active (5). The *K*_m_ of alkylglycerol monooxygenase for tetrahydrobiopterin varies with the assay used, values found were 2.6 μM with the fluorescence-HPLC-based assay (28), 24.6 μM with an UV-based assay (35), and 42 μM with the radiometric assay (15). The *K*_m_ values for the ether lipid substrate were found to be more consistent, 8.9 μM for 1-*O*-pyrenedecyl-glycerol with the fluorescence-HPLC-based assay (28), 11.1 μM for hexadecylglycerol with an UV-based assay (35), and 12 μM for hexadeyclglycerol with the radiometric assay (15).

**FIG 3:**
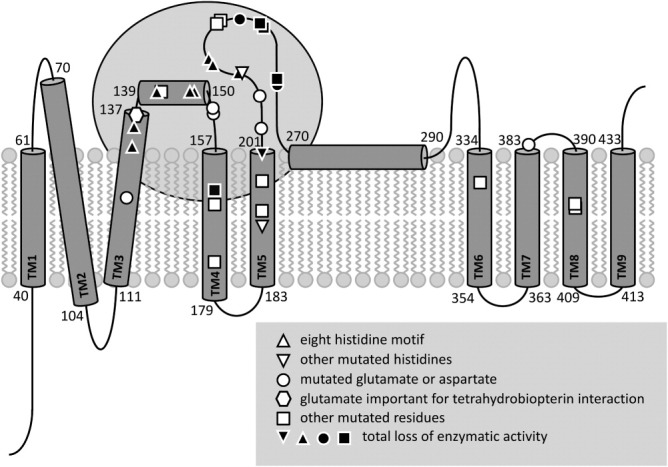
Alkylglycerol monooxygenase topography in the lipid bilayer. Nine transmembrane (TM) and one membrane associated domain have been predicted for the alkylglycerol monooxygenase sequence. Length and positioning of the first five was further refined by *ab initio* modeling using Rosetta membrane (number of starting and end residue of each helix is annotated). Mutations introduced by site directed mutagenesis are shown as symbols: upward directed triangles, eight histidines motif; downward directed triangles, other histidines; circles, glutamates and aspartates; squares, all other mutated residues. Mutations shown with symbols with white lining and black filling all completely abolished alkylglycerol monooxygenase activity. The glutamate at position 137 which has been shown to interact with tetrahydrobiopterin is shown as a hexagon. The potential active site of the enzyme is highlighted in grey. Reproduced with permission, from Watschinger, K., Fuchs, J. E., Yarov-Yarovoy, V., Keller, M. A., Golderer, G., Hermetter, A., Werner-Felmayer, G., Hulo, N., Werner, E. R., *Biochem. J*., 2012, 443, 279–286, © the Biochemical Society.

As the sequence of alkylglycerol monooxygenase is now available it has become possible to switch from rat liver homogenates to homogenates of cells which had been previously transfected with the reading frame of alkylglycerol monooxygenase. This strategy was used in two recent papers, where the effect of strategic mutations in the enzyme on its activity was examined. Thirty four residues were selected, ranging from position 125 to 397 in the 445 amino acids human alkylglycerol monooxygenase protein. This included the eight histidines of the fatty acid hydroxylase motif, three further histidines, five aspartates, four glutamates, five glutamines, four tyrosines, two cysteines, and one tryptophan, asparagine, and arginine (Fig. 3). The respective codons were mutated to alanine codons, an expression plasmid with a cytomegamlovirus promoter transfected to Chinese hamster ovary cells, and the cell homogenates tested for alkylglycerol monooxygenase activity. From all these residues the eight histidines of the fatty acid hydroxylase motif proved to be essential for catalysis (14). In addition mutation of glutamine 162, histidine 201, aspartate 233, asparagines 235, tyrosine 236, tryptophan 243, and aspartate 244 completely abrogated the enzymatic activity (25). Besides the eight histidines of the motif, three additional residues which have been shown to be crucial for catalysis in alkylglycerol monooxygenase (histidine 201, tryptophan 243, and aspartate 244) are found to be conserved in all members of the fatty acid hydroxylases family (14). Another finding was that glutamate 137 adjacent to the second histidine of the fatty acid hydroxylases motif is essential for binding of the cofactor tetrahydrobiopterin as shown by an 18-fold increase in the Michaelis-Menten constant for the cofactor upon mutation to alanine (25). The amino acids around this glutamate have a resemblance to the conserved residues around glutamate 286 in phenylalanine hydroxylase which is involved in tetrahydrobiopterin interaction and yields a 70-fold higher *K*_m_ for tetrahydrobiopterin when mutated to alanine (36, 37). All the important residues needed for ether lipid cleavage and tetrahydrobiopterin interaction clustered together to a cytosolic linker region ranging from residue 132 to 244 of the human protein (Fig. 3).

## STRUCTURE

Concomitant with the unresolved problem to express and purify alkylglycerol monooxygenase protein in its active form is the lack of structural data on the enzyme. As no homologous proteins with a crystal structure are known, a structure based on homology modeling could not be calculated. An *ab initio* structure prediction of the human protein was therefore attempted. Transmembrane region prediction software suggested nine transmembrane helices and an additional membrane associated domain (25). The Rosetta membrane protein prediction tools suggested a structural model for residues 37–205 of the human protein, which has a total length of 445 amino acids. The model yielded five membrane spanning segments and an additional nonmembrane associated helix and is in good accordance with the results obtained from the transmembrane prediction tools. All essential residues identified by site directed mutagenesis which are contained in this region—including five histidines of the eight-histidine motif and the glutamate involved in tetrahydrobiopterin binding—nicely cluster together (25) forming the potential active site of the enzyme.

## OUTLOOK

While it may be a technically challenging task to work on biochemistry and structure of the pure protein due to its exceptional instability, description of a physiological role should now be possible due to the availability of the alkylglycerol monooxygenase sequence and its occurrence in common model organisms such as zebrafish and the nematode *C. elegans*.
